# Mitotic and Proliferative Indices in WHO Grade III Meningioma

**DOI:** 10.3390/cancers12113351

**Published:** 2020-11-12

**Authors:** Andrea Daniela Maier, Christian Beltoft Brøchner, Jiri Bartek Jr., Frank Eriksson, Heidi Ugleholdt, Helle Broholm, Tiit Mathiesen

**Affiliations:** 1 Department of Neurosurgery, Copenhagen University Hospital, Rigshospitalet, Inge Lehmanns Vej 6, 2100 Copenhagen, Denmark; jiri.bartek@sll.se (J.B.J.); tiit.illimar.mathiesen@regionh.dk (T.M.); 2 Pathology Department, Copenhagen University Hospital, Rigshospitalet, Inge Lehmanns Vej 7, 2100 Copenhagen, Denmark; christian.beltoft.broechner@regionh.dk (C.B.B.); heidi.ugleholdt@hotmail.com (H.U.); helle.broholm@regionh.dk (H.B.); 3 Department of Neurosurgery, Karolinska University Hospital, Solnavägen 1, Solna, 17176 Stockholm, Sweden; 4 Department of Clinical Neuroscience and Department of Medicine, Karolinska Institutet, Solnavägen 1, Solna, 17176 Stockholm, Sweden; 5 Section of Biostatistics, Department of Public Health, University of Copenhagen, Øster Farimagsgade 5, 1014 Copenhagen, Denmark; eriksson@sund.ku.dk; 6 Department of Clinical Medicine, University of Copenhagen, Blegdamsvej 3B, 2100 Copenhagen, Denmark

**Keywords:** malignant meningioma, mitotic indices, hot spots, Ki-67, agreement

## Abstract

**Simple Summary:**

Malignant meningiomas are rare primary intracranial tumors associated with considerable morbidity and mortality. The diagnosis is based on the number of mitotic figures (mitotic index, MI). Consequently, the quantification of mitotic figures is prone to inter- and intraobserver variability. The mitotic marker, phosphohistone-H3 (PHH3), has been shown to be a more robust mitotic marker. Despite the prognostic value of MI across all meningioma grades, little is known of the prognostic value of the MI within malignant meningioma. Therefore, this study investigates the MI in a series of malignant meningiomas to analyze the association to progression-free survival and mitotic and proliferative indices. Furthermore, we investigated the precision (repeatability) of mitotic counts and the agreement between MI and PHH3 MI.

**Abstract:**

Meningiomas with inherently high mitotic indices and poor prognosis, such as WHO grade III meningiomas, have not been investigated separately to establish interchangeability between conventional mitotic index counted on H&E stained slides (MI) and mitotic index counted on phosphohistone-H3 stained slides (PHH3 MI). This study investigates the agreement of MI and PHH3 MI and to analyze the association of progression-free survival (PFS) and MI, PHH3 MI, and the proliferative index (PI, Ki-67) in WHO grade III meningioma. Tumor specimens from 24 consecutive patients were analyzed for expression of Ki-67, PHH3 MI, and MI. Quantification was performed independently by two observers who made replicate counts in hot spots and overall tumor staining. Repeatability in replicate counts from MI and PHH3 MI was low in both observers. Consequently, we could not report the agreement. MI, PHH3 MI and hot spot counts of Ki-67 were associated with PFS (MI hot spot HR = 1.61, 95% CI 1.12–2.31, *p* = 0.010; PHH3 MI hot spot HR = 1.59, 95% CI 1.15–2.21, *p* = 0.006; Ki-67 hot spot HR = 1.06, 95% CI 1.02–1.11. *p* = 0.004). We found markedly low repeatability of manually counted MI and PHH3 MI in WHO grade III meningioma, and we could not conclude that the two methods agreed. Subsequently, quantification with better repeatability should be sought. All three biomarkers were associated with PFS.

## 1. Introduction

WHO grade III meningiomas are rare primary intracranial tumors associated with considerable morbidity and mortality. Only 3–5% of meningiomas are malignant (WHO grade III), but exhibit aggressive behavior with an 80% recurrence rate within five years after resection [1]. The diagnosis is based on the number of mitotic figures, the mitotic index (MI), counted by the pathologist on H&E stained slides according to the WHO 2016 grading criteria [2]. Frank anaplasia, papillary and rhabdoid morphology is used to diagnose a minor subset of malignant meningioma cases. Consequently, the quantification of mitotic figures is prone to inter- and intraobserver variability. Mitotic index counted on Phosphohistone-H3 stained slides (PHH3 MI) has been suggested as a reliable and valid surrogate marker of conventional H&E MI with a high intra- and interobserver reproducibility across all meningioma grades [3]. Phosphorylation of histone H3 appears in condensed chromatin, which is expressed in actively dividing cells during the M-phase [4] and appears to improve the identification of mitotic figures [3,5,6]. Despite the diagnostic potential of PHH3 MI, interchangeability between PHH3 MI and conventional H&E mitotic index (MI) has not been investigated within malignant meningioma that inherently harbor a high number of mitoses. Previous studies have investigated the correlation between MI and PHH3 MI [3,7] and established prognostic impact among all meningioma [5] grades, but not elucidated repeatability (precision) and agreement between the methods in a clinical setting.

In addition, the proliferative marker Ki-67 (proliferative index, PI) is widely used, but has shortcomings as a predictive biomarker [5,8,9]. Heterogenous patterns of mitoses, Ki-67, and PHH3 are recognized by pathologists [10,11,12] as a factor to consider when reporting the MI and the PI; they vary with tumor heterogeneity [13,14,15]. However, the PHH3 MI, and PI are strong prognostic parameters in unselected cohorts of meningiomas [6,16], but their significance within the sub-group of malignant meningiomas is controversial [7,17].

Therefore, we investigated the MI, PHH3 MI, and PI in a series of malignant meningiomas to investigate the association of MI and progression-free survival and to study the agreement between MI and PHH3 MI. In addition, we described intratumoral heterogeneity and the proliferative pattern in malignant meningioma.

## 2. Results

### 2.1. Repeatability and Agreement of Conventional MI and PHH3 MI

**Figure 1:**
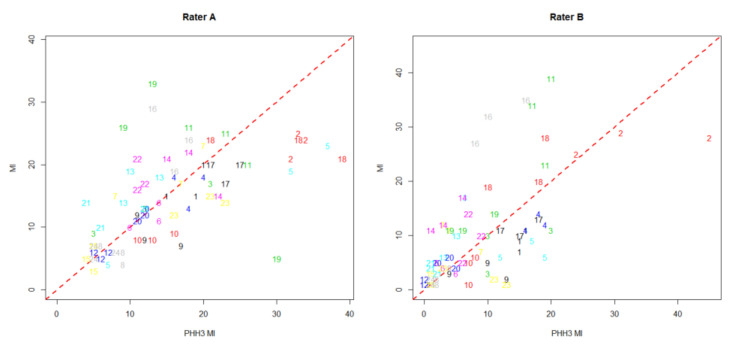
Scatter plot of counts from observers A and B where replicated are represented by the same number.

Figure 1 shows the scatter plots of all counts from observers A and B. The limits of internal agreement (LoiA) for the ratio of single measurements counted by observer A were wide for MI and PHH3 MI and even wider for observer B (Table 1). The limits of agreement for the ratio between MI and PHH3 MI were similarly wide for observer A. These limits were not much wider than the internal limits of agreement; the lack of agreement between the two methods can be explained by lack of repeatability in observer A. In observer B, the wide limits of agreement between MI and PHH3 MI could partly be explained by low repeatability as they were wider than the LoiA (Table 1).

There was no systematic difference between MI and PHH3 MI as the geometric PHH3 mean count was 2.7% higher (*p* = 0.67) for observers A and 10.2% (*p* = 0.52) lower for observer B compared with the geometric MI mean count.

### 2.2. Association of Mitotic and Proliferative Markers and Progression-Free Survival

Cox regression analysis adjusted for age revealed that the mitotic indices counted conventional-ly and with PHH3 in both 10 HPF and 1 HPF were significantly inversely associated with PFS when using the consensus dataset from both observers. The proliferative marker Ki-67 counted in hot spots (per 100 cells), and an overall Ki-67 > 10% were both significantly inversely associated with PFS (Table 2).

### 2.3. Proliferative Patterns and Mitotic Indices in Malignant Meningioma

We observed two different types of proliferative patterns. We observed a heterogenous formation with Ki-67 positive cells tightly packed among negatively stained cells (hot spots shown in Figure 2A) or a homogenously distributed proliferative pattern (Figure 2B). No samples showed a pure hot spot formation or pure homogenously distributed positive cells. We observed a mix of heterogenous and homogenous patterns within the whole-section tumor samples (Type 1 and 2 tumors in Figure 2). Thirteen (54%) tumors had distinct regions of spot patterns combined with other regions with a more homogenous pattern (Type 1 pattern). Eleven (46%) tumors had a gradual transition from proliferative centers to homogenous staining (Type 2 pattern). Examples of the proliferative patterns in all 24 cases can be seen in Supplementary Materials, Figures S1–S24).

We found significantly higher PI and hot spot MI in anaplastic meningiomas compared with papillary and rhabdoid meningiomas (Kruskal-Wallis *p*-value 0.035 and 0.034, respectively). We could not conclude that there was a difference between the histological subgroups in the PHH3 overall or hot spot quantification. Table 3 shows the quantification within the three histological subgroups.

## 3. Discussion

We investigated the MI and PHH3 MI in a series of 24 WHO grade III meningioma to examine the interchangeability between MI and PHH3 MI in tumors with high mitotic indices. We report low repeatability in repetitive counts from two observers. Consequently, we found a lack of agreement between MI and PHH3 MI. We found a significant association between shorter PFS and high Ki-67 and PHH3 MI regardless of hot spot or overall quantification. In addition, we described the proliferative pattern in malignant meningioma and characterized proliferative hot spot formations within the tumor landscape.

### 3.1. Repeatability and Agreement in Quantification of Mitotic Indices

The 2016 WHO grading system has been criticized for the evaluation of the MI counted on H&E stained slides being influenced by difficulties in identifying mitotic figures, confusion with other chromatin changes, and subjective selection of the highest mitotically active area (mitotic hot spots). The mitosis specific marker PHH3 has been suggested to be a robust biomarker with minimal interobserver and interlaboratory variability [5,18], and mitosis specific thresholds in meningioma have been suggested [3]. Though correlation is easy to show between the two methods [3], agreement between the two methods has not been sufficiently determined, as a high correlation does not imply that two methods agree [19,20]. We found wide limits of internal agreement in both MI and PHH3 investigated by observers A and B. This indicated poor repeatability in both MI and PHH3 MI. The wide limits should not be extrapolated to observers, in general. However, considering our results in a clinical setting, the wide internal limits of agreement could imply grade switching (from grade III to II) or grossly overestimating mitotic counts in WHO grade III tumors.

When repeatability is poor, the agreement between the two methods is bound to be poor [21,22]. In the case of observer A, the limits of agreement for the ratio between MI and PHH3 MI were almost the same as the LoiA, and the poor agreement could be explained by low repeatability. In the case of observer B, the wide LoiA only partially explained the poor agreement (the limits of agreement for the ratio between MI and PHH3 MI: 85–454%), implying that agreement in the case of observer B was indeed poor. For both observers, the repeatability was poor, and we could, therefore, not conclude that the methods agreed.

Our results are unprecedented for two reasons. First, the methodology in previous studies had focused on a correlation between the MI and PHH3 MI [3] and establishing the prognostic impact of PHH3 [5]. Though previous studies report low interobserver variability of PHH3 in uveal melanoma [18] and meningioma [3]; repeated counts were not performed. To our knowledge, repeated counts of mitotic indices have not been previously investigated in meningioma. A previous study in mitotic counts in lymphoma investigated repeated counts for one observer, but did not calculate limits of agreement [23].

The wide LoiA we found in this study should not necessarily be extrapolated to observers, in general, but underlines the importance of reporting repeatability when investigating agreement and interobserver variability. Secondly, a series of meningiomas seldomly include several tumors with high mitotic numbers. Our initial analysis showed that the variability of the replicates increased with the magnitude of the replicates. As only two observers were studied, we are reluctant to make clinical recommendations. However, we would advise extreme caution in making clinical decisions or constructing treatment algorithms based on moderate differences in MI in meningiomas with high MI (WHO grade III), regardless of method, as our results show that the variability increases with the magnitude of the replicates (in both MI and PHH3 MI). Our results still show an association with PFS, but this is based on our consensus dataset. In cases where a precise MI is needed, we would recommend three replicate counts of the same tumor and using the mean of these replicate counts. Taken together, our results imply that tumors with high mitotic indices have a substantial uncertainty bound to their reported MI. In addition, MI and PHH3 MI analyzed in a clinical setting have low repeatability.

### 3.2. Association of Mitotic and Proliferative Indices and Progression-Free Survival in WHO Grade III Meningioma

**Figure 2:**
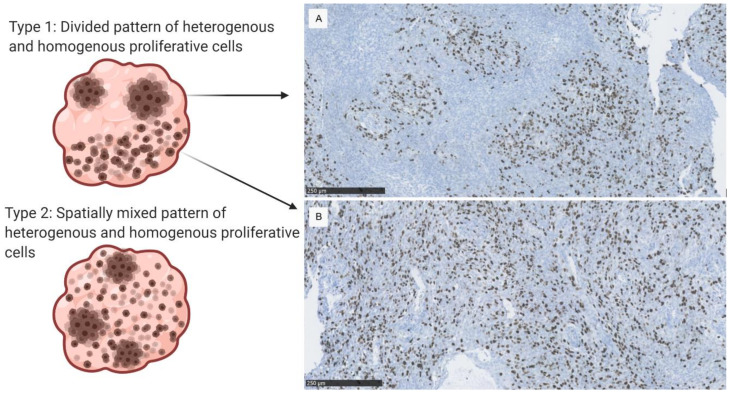
Anaplastic meningioma from the cohort, Ki-67 stain. (**A**) Hot spot pattern with distinct areas of Ki-67 positive cells. (**B**) A homogenous proliferative pattern. The A and B patterns can be mixed within the tumor resulting in either a type 1 or type 2 proliferative pattern (shown on the left), which were observed in the 24 malignant meningioma samples.

For assessing the association of the PI, MI, and PHH3 MI and progression-free survival, we used the consensus data set and the average of the repeated counts from the two observers. We established that proliferative indices and mitotic indices in hot spots and in overall tumor staining were associated with progression-free survival in grade III meningioma. We did not find a characteristic homogenous hot spot pattern that would provide a characteristic phenotype of the respective histological sections; instead, we observed regionally heterogenous hot spot patterns with either abrupt or smooth transition to areas with a more homogenous proliferative staining in the tumors. Hence, we have suggested terminology to classify hot spot patterns (Figure 2), which may be helpful for further studies that link heterogenous visual appearance to functional subspecialization and prognosis. Emerging genotypical prognostic factors, such as the TERT promoter mutation, have shown both spatial and temporal heterogeneity in high grade and malignant meningioma [24,25]. Extensive molecular profiling of meningiomas has identified clinically relevant subgroups based on genomic, transcriptomic (including average transcript levels of MKI67), and epigenomic analyses within WHO grade I and II meningiomas [26,27], but grade WHO grade III tumors have not undergone such extensive analysis yet. Our report of substantial phenotypical proliferative heterogeneity within the tumor landscape underlines the necessity of considering spatial heterogeneity in future molecular analyses. Proliferative hot spots may either represent random phenotypical pathomorphological features with or without additional prognostic significance, or they could reflect subspecialized biological functions within the tumor. Finally, they could even represent centers of progression from where a malignant pattern originates. Hot spot quantification did not necessarily add prognostically relevant information compared with overall quantification in our material of WHO grade III meningiomas (Table 2). Larger materials may still reveal a difference, but we did not observe a difference in the PFS association between overall mitotic quantification and hot spot quantification, as observed in melanoma and adrenocorticoid cancer [14,15]. Interestingly, Focke et al. [13], found that heterogenous PI in early-stage breast cancer exceeded the variability between individual tumors, and Stålhammer et al. [28] concluded that Ki-67 hot spots should be the best marker for proliferation in breast cancer. Such findings argue for the importance of regional heterogeneity in some tumors. Our analyses did not specifically target the biological function of hot spots; hence, the function of oncological intratumoral subspecialization remains un-answered.

Our results showed a significantly higher number of mitoses and Ki-67 positive cells in anaplastic meningioma compared with rhabdoid and papillary meningioma. This is not a novel finding—it corroborates our earlier finding of anaplastic meningioma patients having shorter PFS than papillary and rhabdoid meningioma patients [17]. Furthermore, molecular data strengthen the hypothesis that rhabdoid and papillary meningioma are different clinical and biological entities [29].

### 3.3. Study Limitations

The retrospective nature of this study had the limitations of all such studies. Though visual assessment was performed by two researchers, independent of each other’s results and earlier quantification, no digital evaluation was performed to corroborate the findings. Though 24 is a small sample size, and the conclusions must be seen in the light of this, the cohort is relatively large when considering the rarity of malignant meningioma. The strength of our study is the methodological aspect of mitotic quantification in tumors that inherit high mitotic numbers.

## 4. Materials and Methods

### 4.1. Patient Data and Clinical Parameters

We recently described clinical outcomes in a cohort of 24 consecutive patients who underwent surgical treatment for WHO grade III meningiomas at the department of Neurosurgery and diagnosed at the Pathology department at Rigshospitalet from December 2000 to March 2014 [17]. This study comprises extensive immunohistochemical analyses of the cohort reported in our previous paper [17]. Progression-free survival was defined (PFS) as the time from initial surgery of a grade III meningioma to radiological progression, reappearance of tumor, or death from any cause. Thirteen (54%) of the 24 patients were women. The end of follow-up was in March 2020. The median potential follow-up time after the first WHO grade III surgery was 126 months (range 73–282 months). Twenty-one had a recurrence during follow-up; only one patient had not died or experienced a recurrence. Sixteen (67%) meningiomas were anaplastic, four (17%) rhabdoid, and four (17%) papillary.

### 4.2. Histological Features, WHO-Grading and Mitotic Index

The H&E (Hematoxylin and Eosin) whole slide tissue stains were reviewed and classified according to the WHO 2016 classification [2] by a senior neuropathologist (HB). Initial consensus on staining interpretation was undertaken with HB prior to MI, PHH3, and Ki-67 evaluation by two independent observers; one junior doctor and one specialist registrar in pathology. The two independent observers (observers A and B) calculated MI, PHH3, and Ki-67 indices as whole section averages (overall staining) and in hot spots. Observers A and B repeated quantification for all indices three times each. Each assessment was blinded from previous assessments and the other assessor; these data were used to assess repeatability (internal agreement) and agreement between MI and PHH3 MI.

In cases with large discrepancies (±15%), slides were re-evaluated, and the consensus was reached. The means from all six counts in the consensus dataset were used for the survival analyses and for reporting mean counts in the histological subgroups.

### 4.3. Immunohistochemistry

Formalin-fixed paraffin-embedded (FFPE) tissue blocks were cut into 4 µm sections and incubated with mouse anti-human Ki-67 antibody (clone MIB-1 dilution 1:50, Agilent GA626 Dako Denmark A/S, Glostrup) or rabbit polyclonal anti-PHH3 antibody (clone Poly dilution 1:250, Cell Marque, 369A-16, Darmstadt, Germany). An automated immunostainer (Dako Omnis, Agilent, Santa Clara, CA, United States) was used. For Ki-67 immunostaining tissue samples were pre-heated (30 min at 97 °C), a peroxidase blocking agent was added, the primary antibody was added for 40 min, and secondary antibody for 20 min. PHH3 staining followed the same procedure with the BenchMark ULTRA automated slide staining system and incubation periods of 20 min with primary and secondary antibodies. Human tonsil tissue was used for controls in each run. All samples were counterstained with hematoxylin.

### 4.4. Assessment of Hot Spot and Overall Tumor Staining

Histopathological hot spots were defined visually as areas with increased density of Ki-67 positive cells compared to the surrounding tumor tissue. Ki-67 hot spot index was defined as the percentage of positively stained tumor cell nuclei in 100 manually counted cells in a visually selected area with the highest density of immunoreactivity in each slide. The overall Ki-67 staining was assessed in the full section at 20–50× magnification. The results were grouped as 0–10%, 10–25%, 25–50%, and >50% positive cells. PHH3 MI and MI were calculated as the highest number of mitotic figures in 10 consecutive HPF (high power field, 400× magnification). The PHH3 hot spots and mitotic hot spots were quantified as the maximum number of mitotic figures observable (a hypermitotic area) in one HPF.

### 4.5. Statistical Analysis

We analyzed repeatability (precision) of observers A and B separately and agreement between MI and PHH3 MI before the second quantification of slides with more than ±15% discrepancy to reach consensus. The counts were visualized with scatter plots. Our initial analysis showed the variability increased with the magnitude of the replicates (count number 1–3 for both observers separately). After a log-transformation, the variability was reasonably stable. Observer B observed zero mitoses in one tumor (all three replicate counts had the value of 0). The three data points were changed from 0 to 1 in order to allow for the log-transform. We repeated this procedure, but changed the value to 0.5 and then 0.1. This did not change the conclusions from the analysis. We investigated repeatability by calculating the limits of internal agreement (LoiA) of the ratio of single measurements for each observer separately. These limits estimate the interval in which 95% of ratios between replicates are expected to lie. To assess the agreement of MI and PHH3 MI counts, we calculated the limits of agreement for the ratio of single counts between the two methods. That is, we estimated the 95% reference interval for the ratio of one MI and one PHH3 MI count from the same observer. We calculated the limits of agreement for single measurements, and not the average of the replicates, as using single measurements in clinical practice [21,22]. The internal limits of agreement and the limits of agreement between methods were calculated as suggested by Bland and Altman (1999, Section 4 and Section 5, respectively) [21].

For the survival analysis, the consensus data set was used, and the means from both observers’ three counts were used. The survival analysis was done using Cox proportional hazard regression analysis. MI and PHH3 MI are continuous variables. Ki-67 was dichotomized using a pre-hoc cut-off of 10%. All hazard ratios were adjusted for age. A p-value of less than 0.05 was considered significant. Statistical analysis was done with the open-source software ‘R’ version 3.4.1 (R foundation, Vienna, Austria).

### 4.6. Ethical Approval

This study was approved by the Danish Regional Ethics Committee (approval number H-6-2014-010) on 10 April 2014. The need for patient consent was waived.

## 5. Conclusions

We performed an extensive analysis of the repeatability of mitotic indices counted conventionally on H&E stained and PHH3 stained slides by two observers in WHO grade III meningioma; we report a poor internal agreement. Consequently, the agreement between the H&E and PHH3 mitotic quantification methods was also poor in WHO grade III meningiomas. We found two types of proliferative patterns in malignant meningioma, both including hot spots formations. We found a statistically significant association with progression-free survival and Ki-67, quantified in hot spots, and overall staining. The association was also significant in mitotic indices; both in hot spots and in overall staining of conventional H&E and PHH3.

## Figures and Tables

**Table 1:** The internal limits of agreement for observers A and B separately. The limits of agreement for the ratio between single measurements of MI and PHH3 MI for each observer separately.

**Repeatability (Limits of Internal Agreement (95% CI) of the Ratio of Measurements)**				
Rater A	MI	63–173%	PHH3 MI	64–178%
Rater B	MI	72–256%	PHH3 MI	70–229%
Agreement between Methods (Limits of Agreement, Ratio between MI and PHH3 MI)				
Rater A		64–192%		
Rater B		85–454%		

**Table 2:** Cox regression analysis of the association between mitotic and proliferative biomarkers and progression-free survival in 24 malignant meningioma patients.

**Overall Staining in Relation to PFS**	**No. of Patients**	**HR (95% CI)**	***p*-Value**
Mitoses in 10 HPF (MI)	24	1.08 (1.01–1.15)	0.0221 *
PHH3 mitoses in 10 HPF (PHH3 MI)	24	1.09 (1.03–1.15)	0.0032 *
Ki-67 overall expression < 10%	9	1.00	
Ki-67 overall expression > 10%	15	5.40 (1.79–16.27)	0.0028 *
Hot Spot Staining in Relation to PFS	No. of Patients	HR (95% CI)	*p*-Value
Max. no. mitoses in 1 HPF	24	1.61 (1.12–2.31)	0.0103 *
Max. no. PHH3 mitoses in 1 HPF	24	1.59 (1.15–2.21)	0.0056 *
Max. no. Ki-67 positive nuclei/100 nuclei	24	1.06 (1.02–1.11)	0.0040 *

The consensus data from observers A and B were used for the analyses. All analyses were adjusted for age. * *p* < 0.05.

**Table 3:** Quantification of conventional mitotic index (MI), PHH3 MI, and Ki-67 in hot spots and overall tumor section.

Continuous Counts	Median, All Types (Range)	Anaplastic *n* = 16	Rhabdoid *n* = 4	Papillary *n* = 4	Kruskal-Wallis ꭓ^2^ *p*-Value
Mitosis overall/HPF ^1^	1.2 (0.3–2.8)	1.4	0.6	0.7	0.049 *
Mitosis hotspot/HPF ^2^	2.8 (1.2–6.2)	3.4	1.8	1.9	0.034 *
PHH3 overall/HPF ^1^	1.1 (0.3–3.3)	1.3	0.7	0.8	0.184
PHH3 hotspot/HPF ^2^	3.0 (1.0–6.3)	3.3	2.3	2.3	0.263
Ki-67 hot spot/100 cells ^4^	31% (8–52%)	34%	18%	18%	0.035 *
Overall Ki-67 Categories	1–10%	10–25%	25–50%	>50%	
No. of samples (% of total) ^3^	9 (38%)	7 (29%)	6 (25%)	2 (8%)	

^1^ PHH3 and mitosis overall counting was determined by counting mitoses in 10 consecutive HPF 400× magnification. ^2^ PHH3 and mitosis hot spot counting was the highest number of mitoses counted in a single HPF 400× magnification. ^3^ Ki-67 Staining estimated by evaluating overall immunoreactivity in 20–50× magnification. ^4^ Ki-67 staining in hot spots evaluated by counting positive cells pr. 100 cells. * *p* < 0.05.

## Supplementary Materials

The following are available online at https://www.mdpi.com/2072-6694/12/11/3351/s1, Figures S1–S24: Case 1–24.
