# Supplementary material for: Mitotic and Proliferative Indices in WHO Grade III Meningioma
Source: Cancers (Basel). 2020 Nov 12;12(11):3351. doi: 10.3390/cancers12113351 (PMC7697885; doi:10.3390/cancers12113351)
Supplement: Supplementary file 1 [file cancers-12-03351-s001.pdf]

Supplementary Materials: Mitotic and Proliferative Indices in WHO Grade III Meningioma

Andrea Daniela Maier, Christian Beltoft Brøchner, Jiri Bartek Jr., Frank Eriksson, Heidi Ugleholdt, Helle Broholm and Tiit Mathiesen

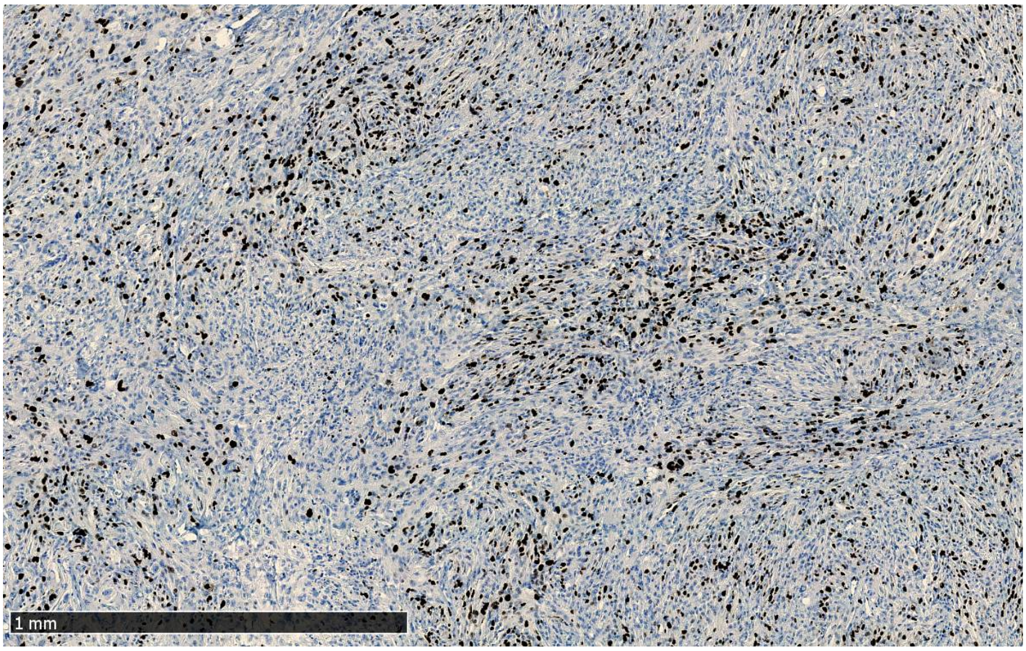

Figure 1. Case 1.

Clear hot spot formation with some Ki-67 positive cells between hot spots in a type 2 proliferative pattern. Though it could look like a type 1 tumor, we see underlying proliferative activity between the hot spot formations. Anaplastic meningioma, WHO grade III. Total magnification ×40.

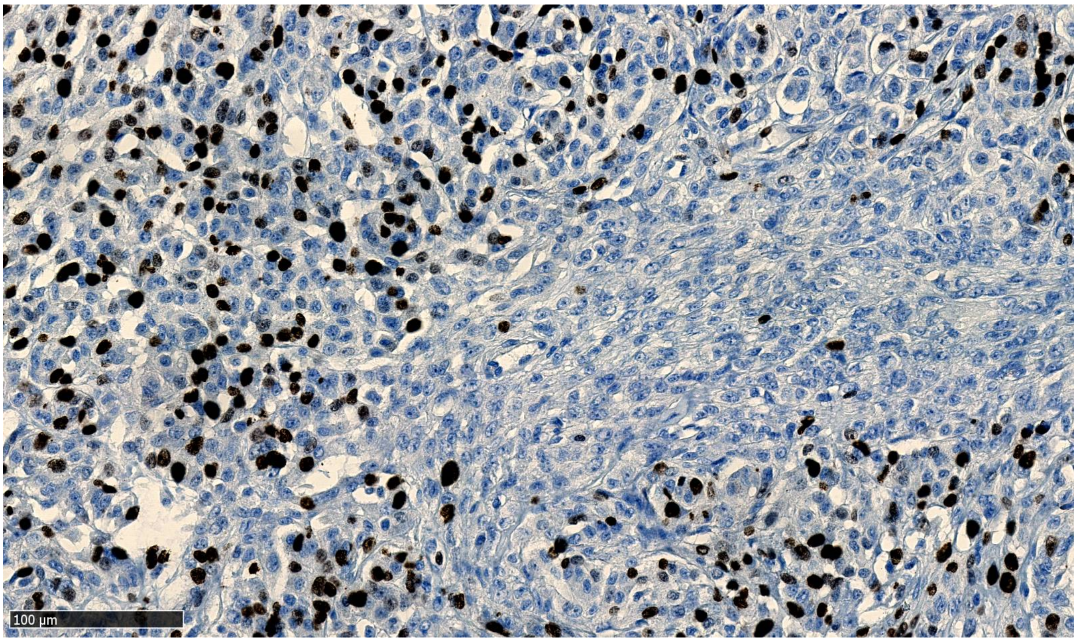

Figure S2. Case 2.

Detail of heterogenous Ki-67 staining in a type 1 proliferative pattern. We see areas with no Ki-67 positivity. Anaplastic meningioma, WHO grade III. Total magnification ×300.

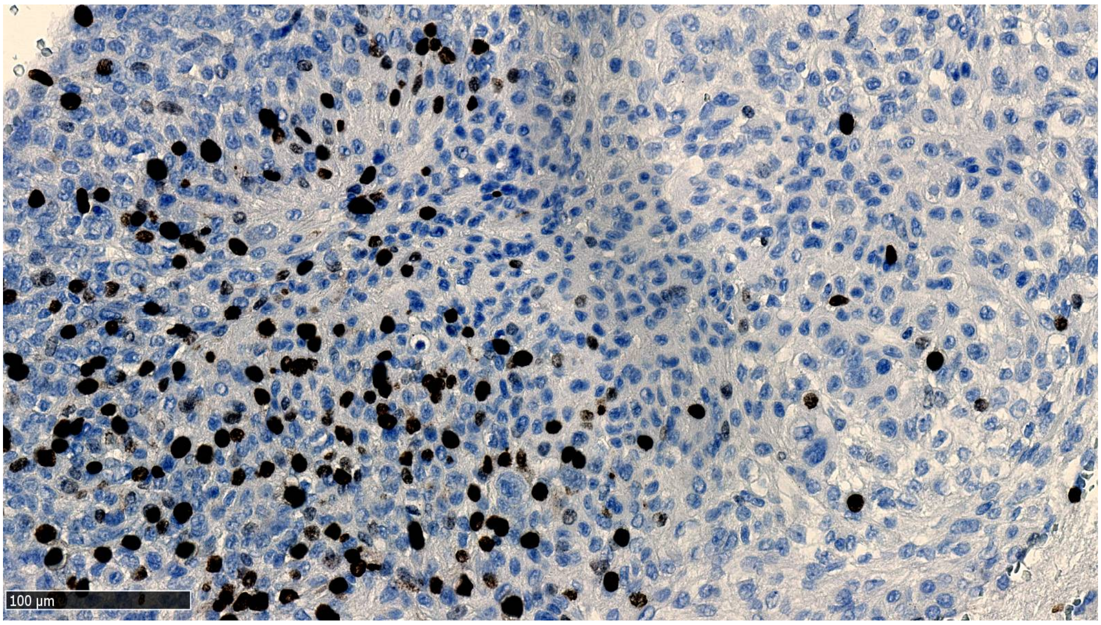

Figure S3. Case 3.

Details of proliferative pattern in a type 1 tumor. A clear 'thinning out' of Ki-67 positive cells across a short distance is seen. Anaplastic meningioma, WHO grade III. Total magnification ×300.

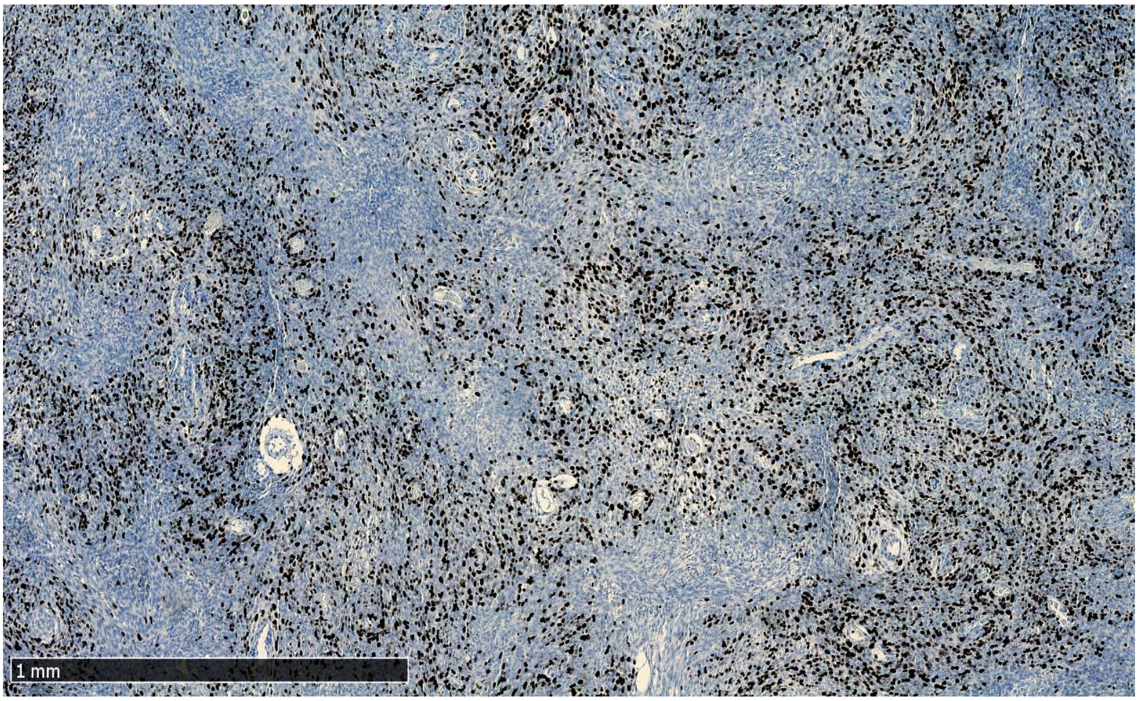

Figure S4. Case 4.

Case 4: Type 1 proliferative pattern, heterogenous area. Same tumor as shown in figure 1. Anaplastic meningioma, WHO grade III. Total magnification ×40.

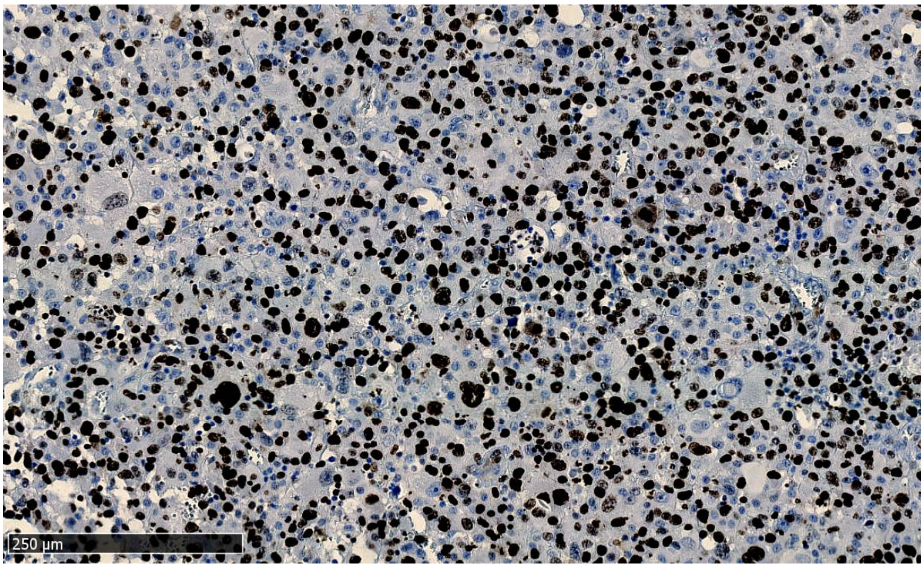

Figure S5. Case 5.

Case 5: Highly proliferative and homogenous area of a rhabdoid meningioma, WHO grade III. A type 1 proliferative pattern was observed. In other areas of the tumor, we saw a clear hot spot pattern. Total magnification ×100.

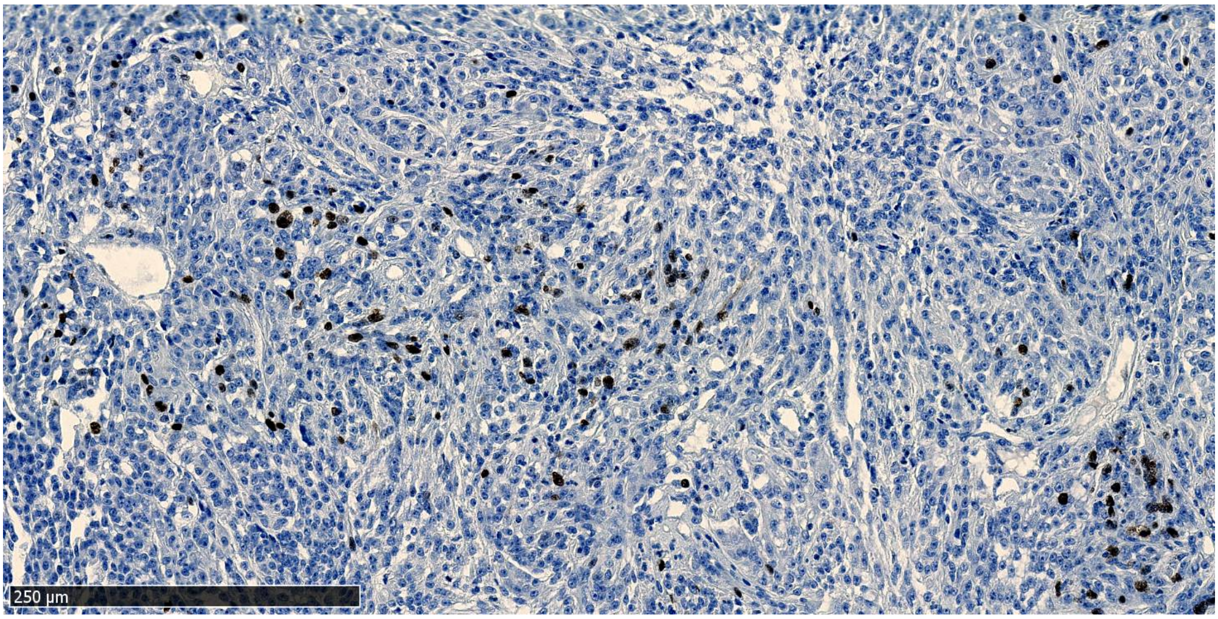

Figure S6. Case 6.

Case 6: Type 1 proliferative pattern with large areas of negative cells in between Ki-67 positive hot spots. This case had extensive lymphocyte infiltration. Rhabdoid meningioma, WHO grade III. Total magnification ×150.

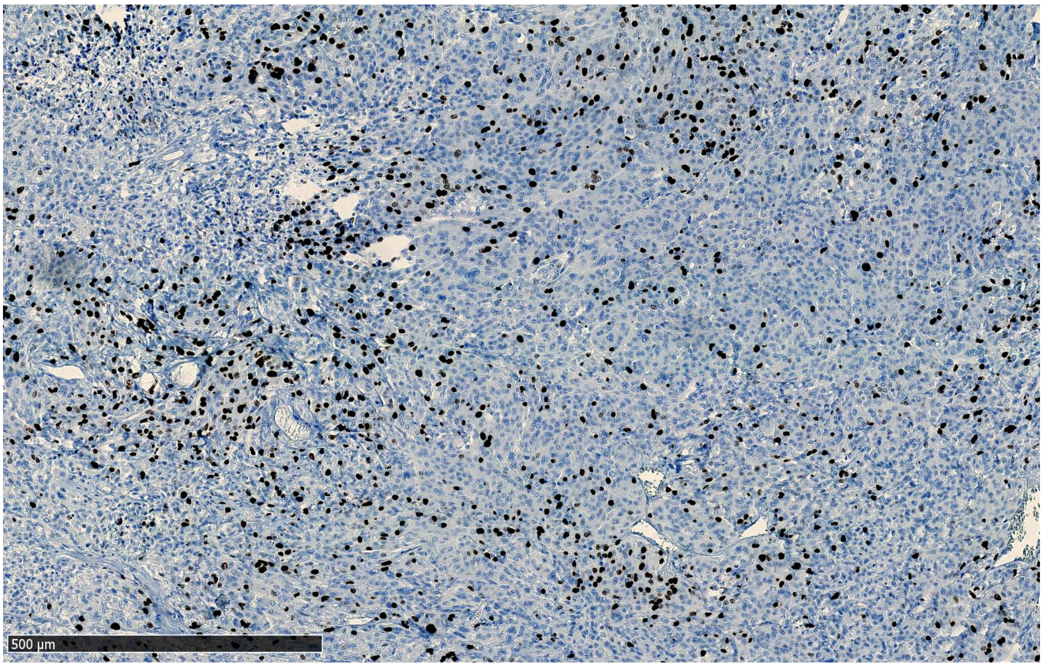

Figure S7. Case 7.

Case 7: Hot spots seen on a 'background level' of proliferative activity: A type 2 proliferative pattern. Anaplastic meningioma, WHO grade III. Total magnification ×90.

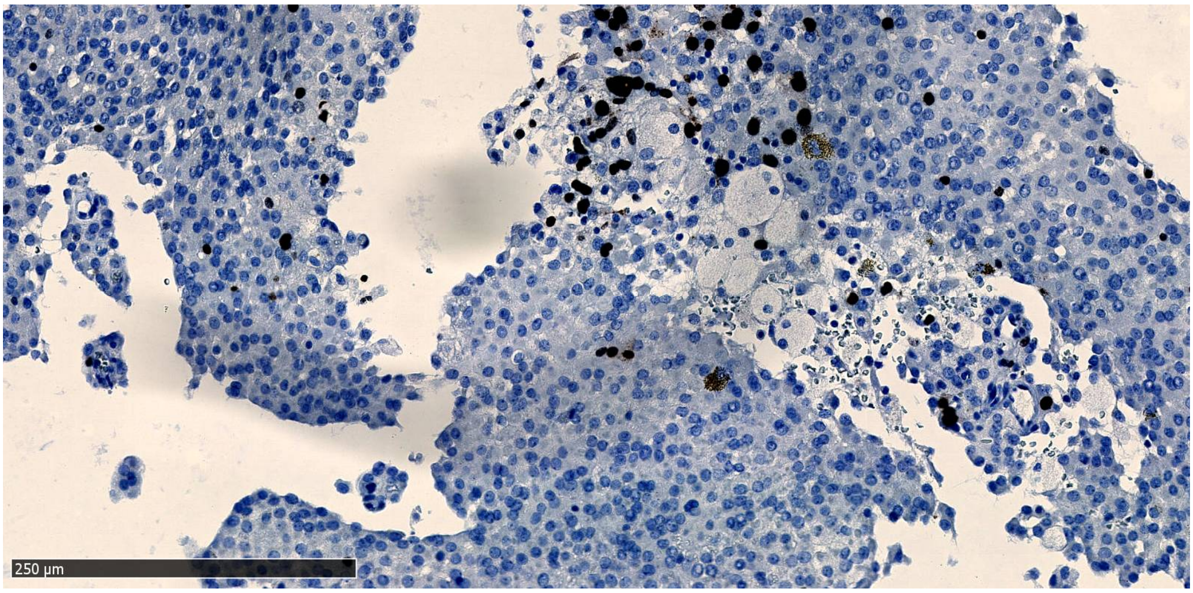

Figure S8. Case 8.

Case 8: Type 1 proliferative pattern in a fragmented sample of rhabdoid meningioma, WHO grade III. Fragmentation makes pattern recognition difficult, but some islands of tumor cells were seen without Ki-67 positive cells. Total magnification ×150.

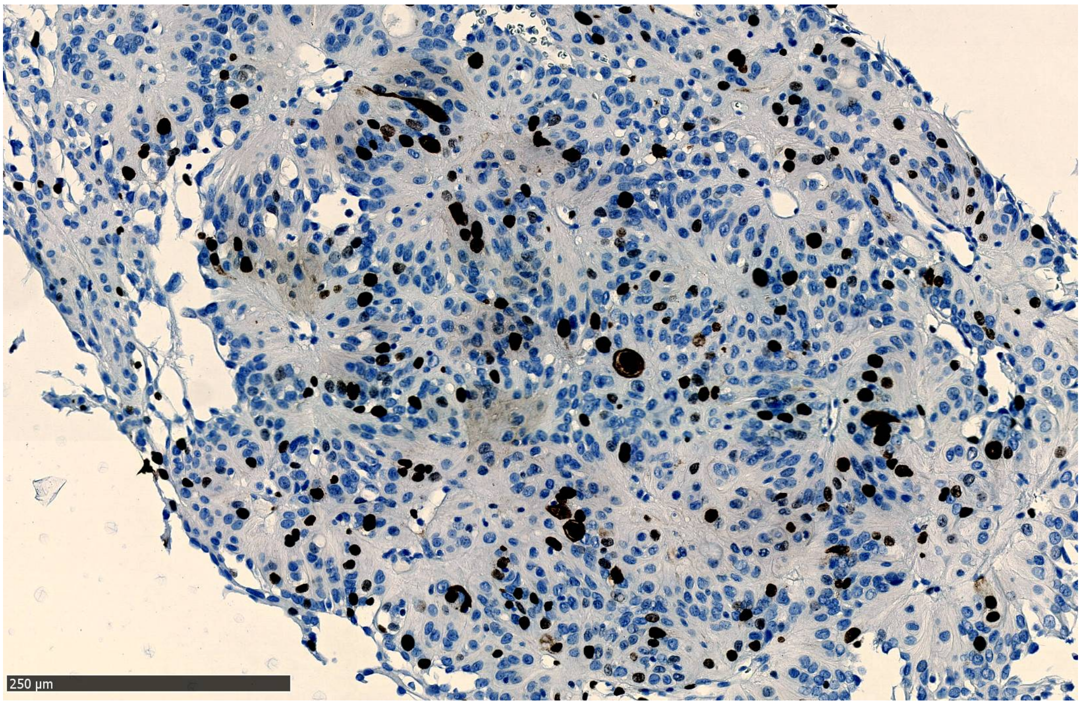

Figure S9. Case 9.

Case 9: More homogenous part of proliferative type 2 papillary meningioma, WHO grade III, but classification of the proliferative pattern was difficult because of the papillary formations. Total magnification ×150.

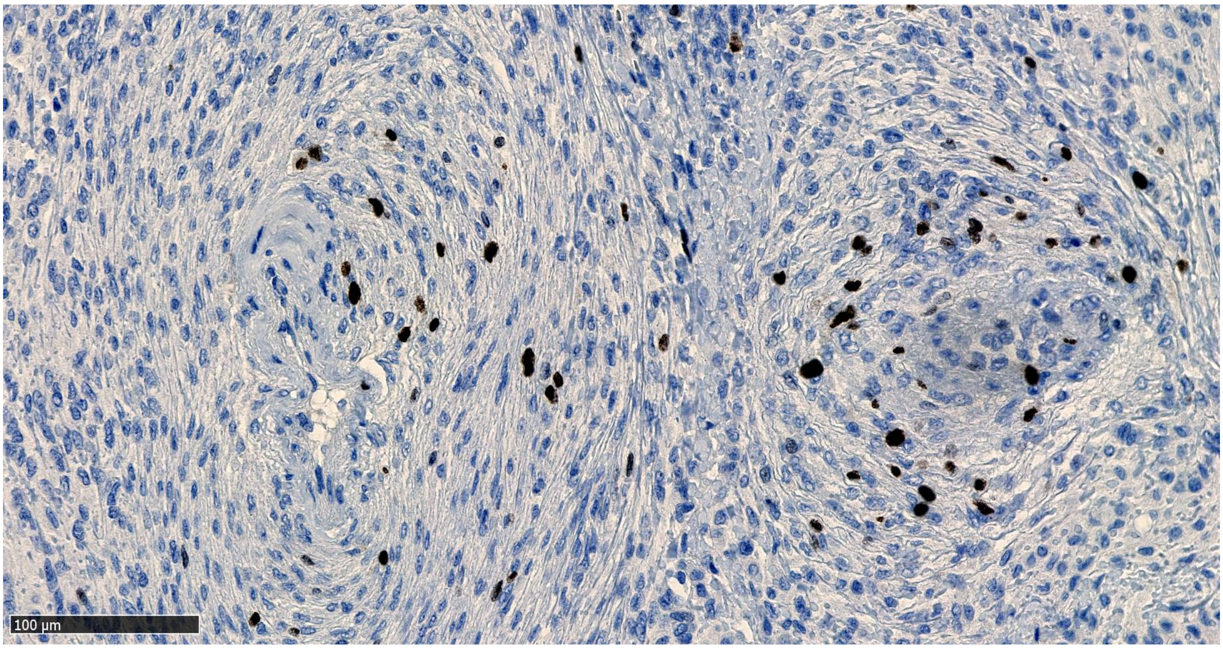

Figure S10. Case 10.

Case 10: Detail of type 1 proliferative pattern, anaplastic meningioma, WHO grade III. Total magnification ×230.

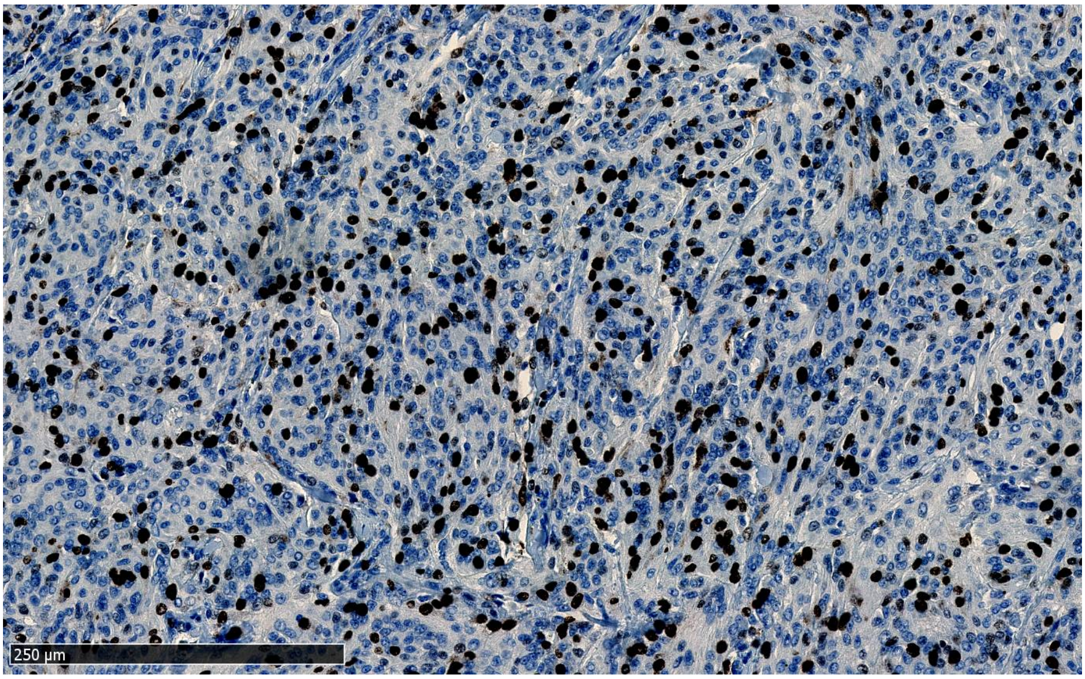

Figure S11. Case 11.

Case 11: Homogenous area of highly proliferative active and cell dense anaplastic meningioma, WHO grade III, type 1 proliferative pattern. Other areas of tumor presented with clear hot spots formations. Total magnification ×150.

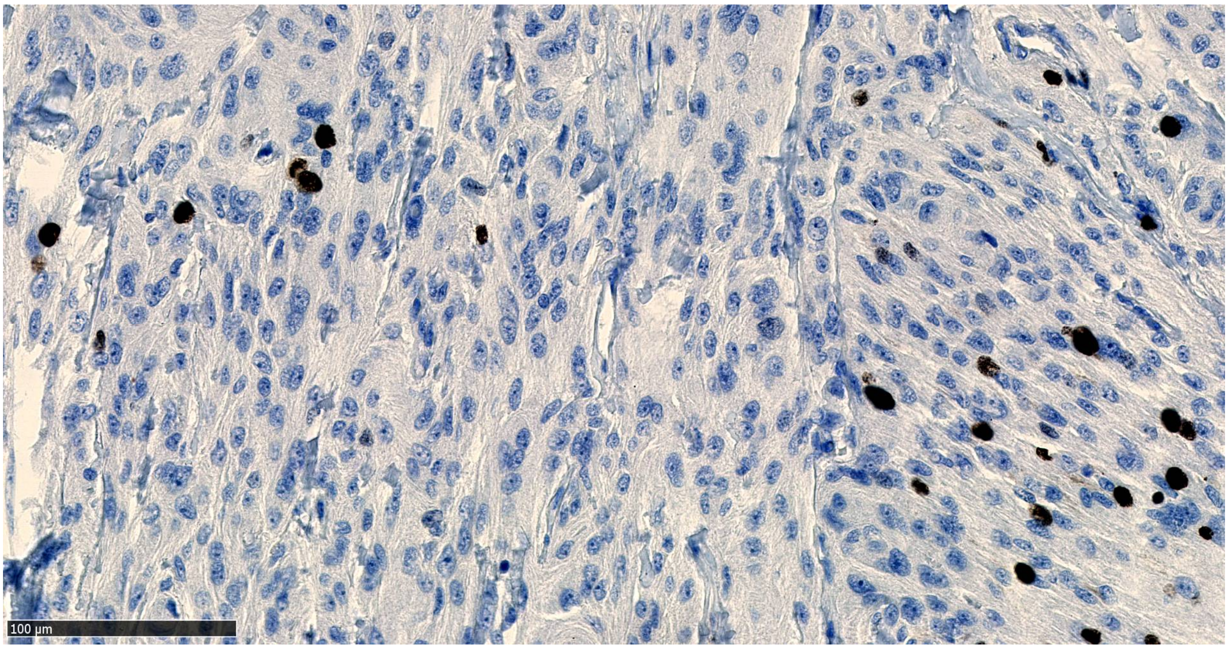

Figure S12. Case 12.

Case 12: Detail of type 1 proliferative pattern in a tumor with relatively sparse Ki-67 positive cells. Anaplastic meningioma, WHO grade III. Total magnification ×300.

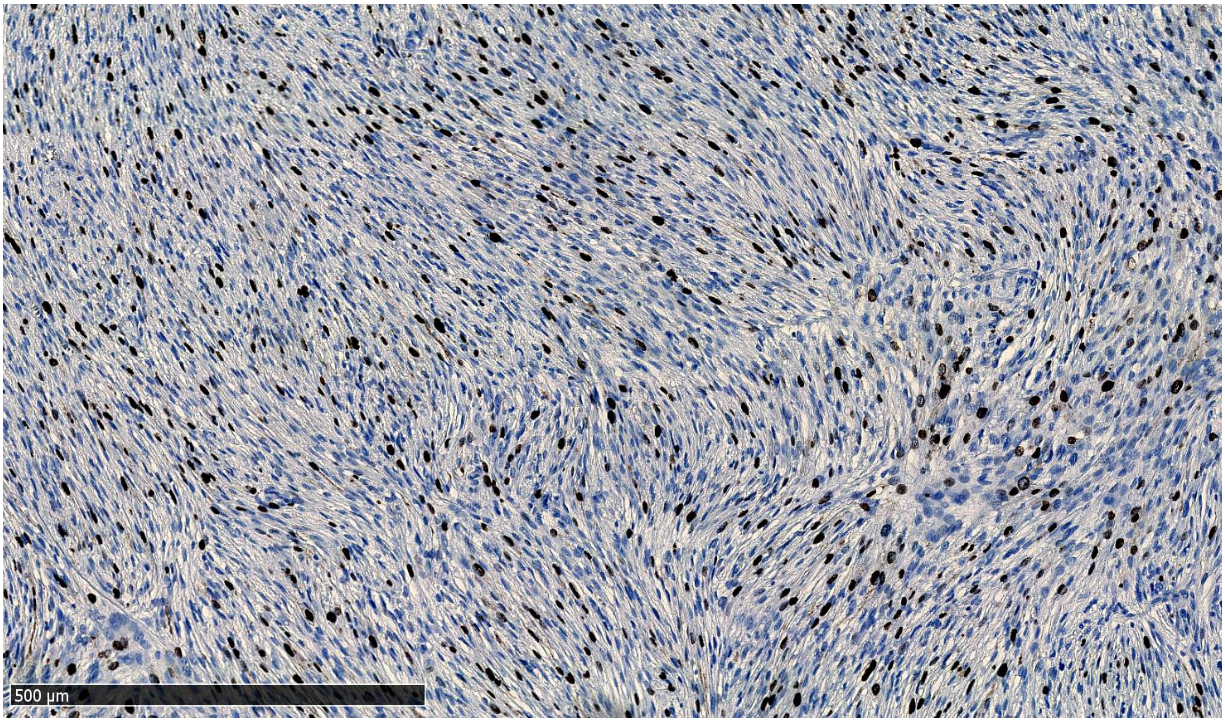

Figure S13. Case 13.

Case 13: Type 2 proliferative tumor. Anaplastic meningioma, WHO grade III. Total magnification ×100.

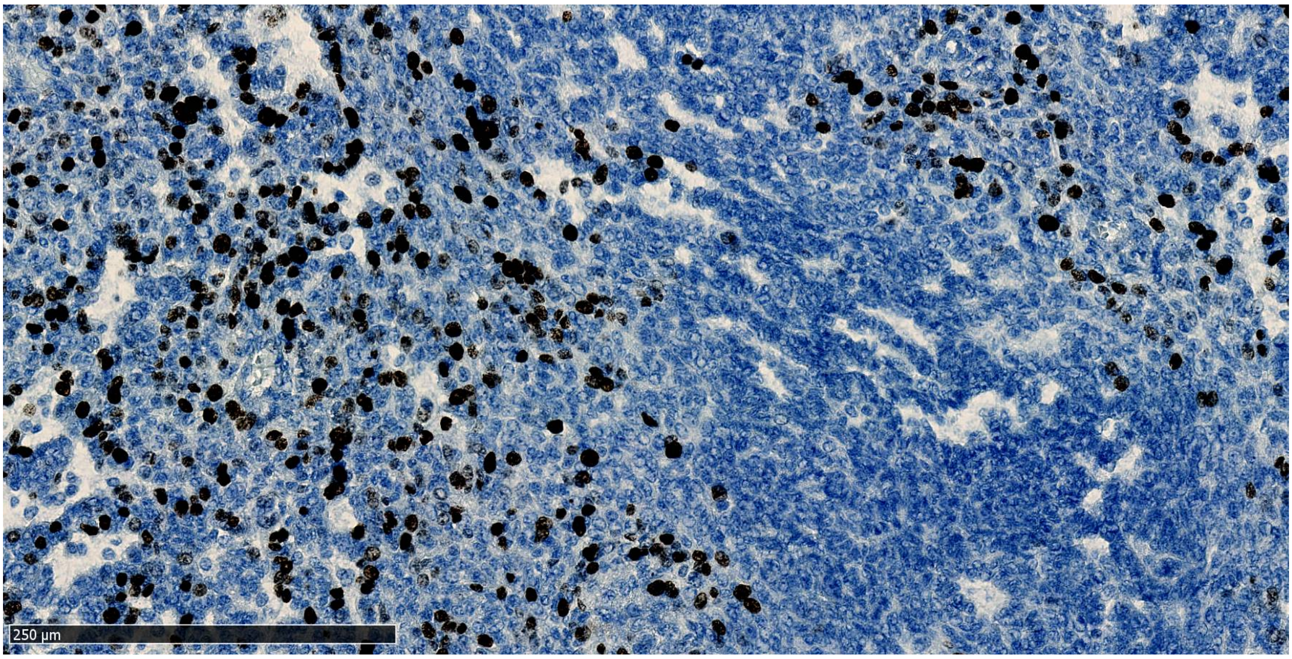

Figure S14. Case 14.

Case 14: Type 1 proliferative pattern, anaplastic meningioma, WHO grade III. Total magnification ×200.

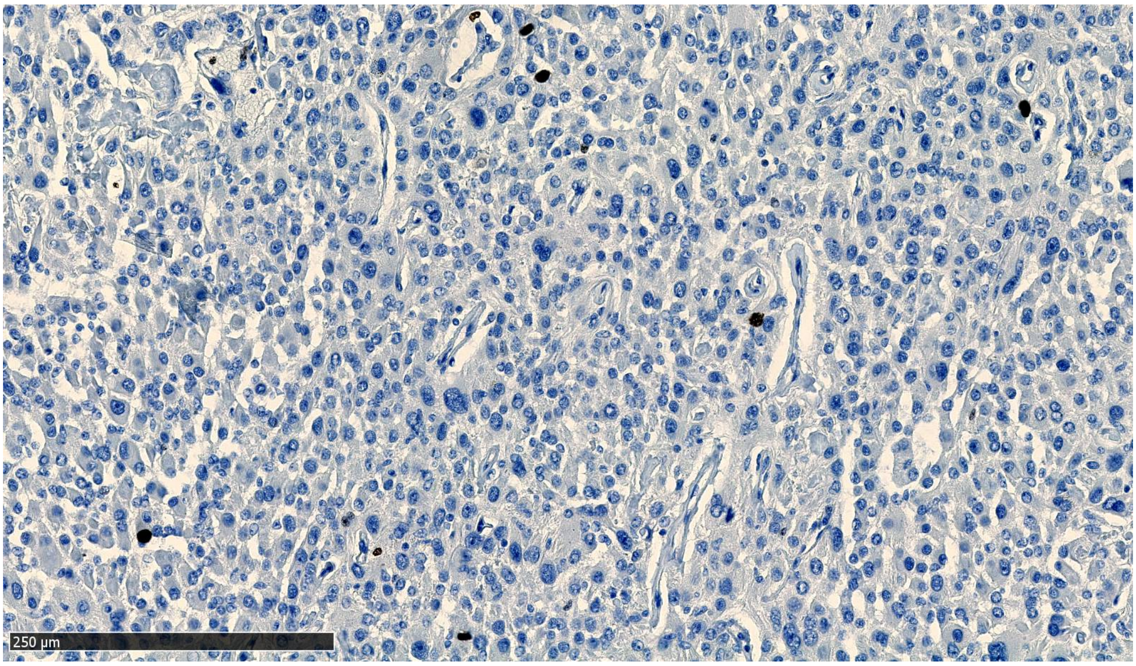

Figure S15. Case 15.

Case 15: Low proliferative index in a rhabdoid meningioma, WHO grade III, with a type 1 proliferative pattern. In other areas small hot spots were observed. In low proliferative index it is difficult to establish proliferation type. Total magnification ×150.

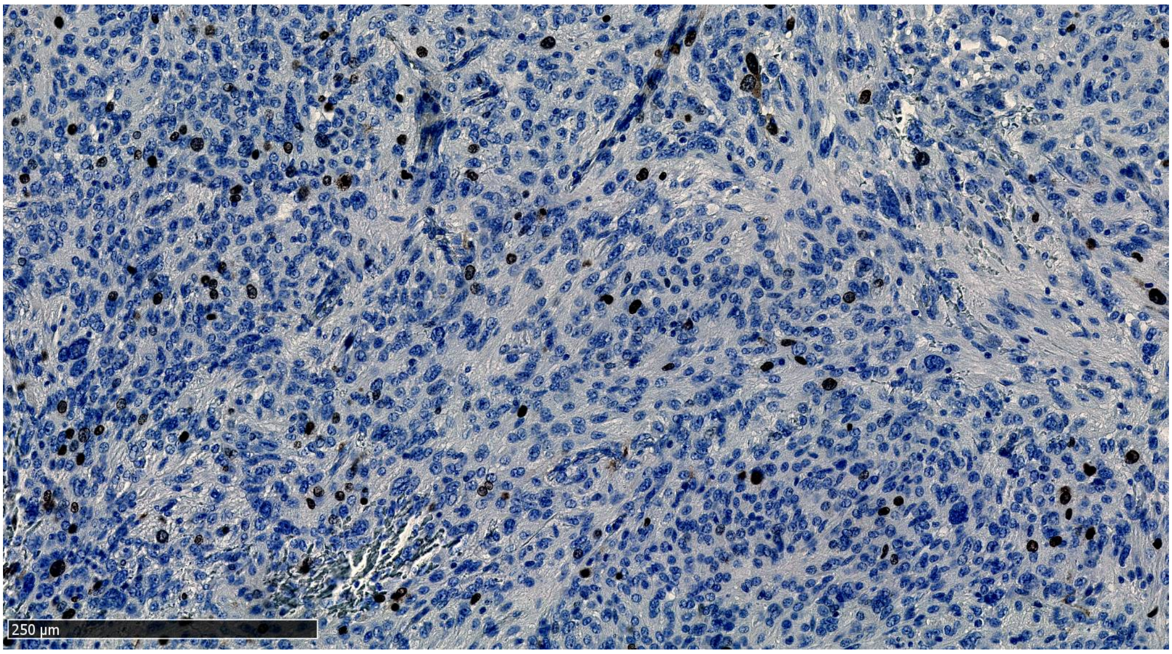

Figure S16. Case 16.

Case 16: Type 2 proliferative pattern between hot spots in an anaplastic meningioma, WHO grade III. Total magnification ×150.

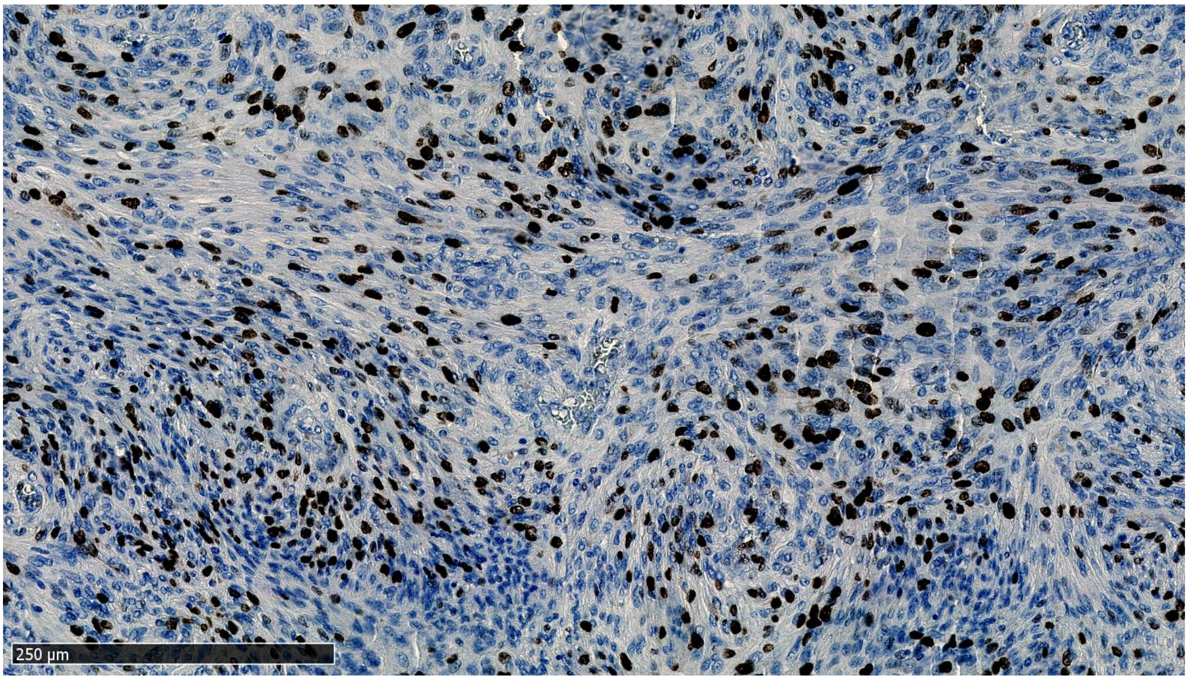

Figure S17. Case 17.

Case 17: Type 2 proliferative pattern. Anaplastic meningioma, WHO grade III. Total magnification ×150.

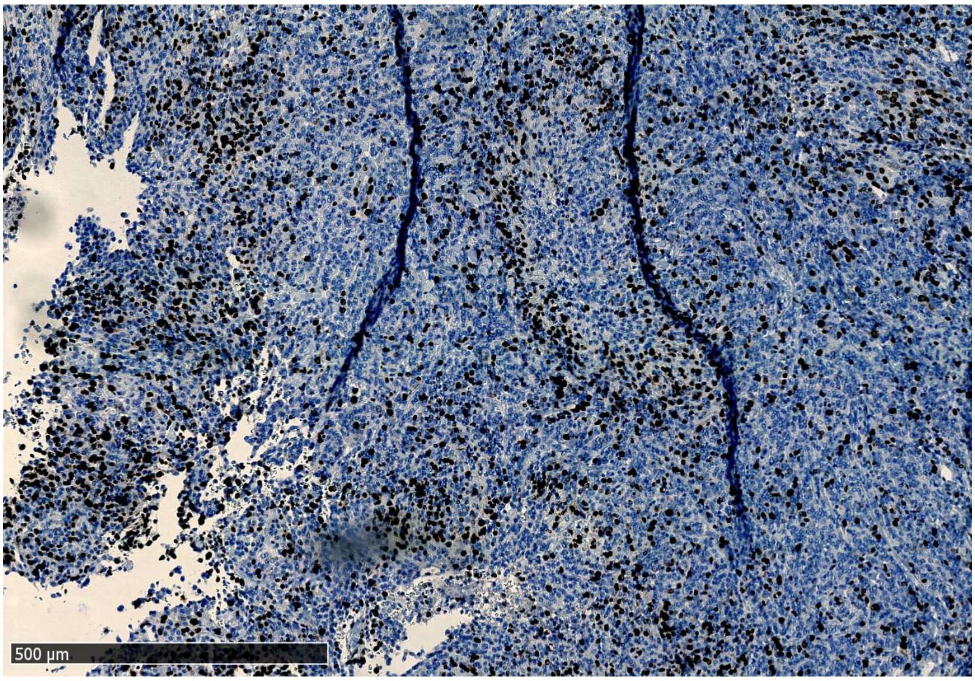

Figure S18. Case 18.

Case 18: Type 2 proliferative pattern, anaplastic meningioma, WHO grade III. Unfortunately, seen with artefactual folding and a rather thick slide. Hot spot areas in an otherwise type 2 proliferative pattern. Total magnification ×70.

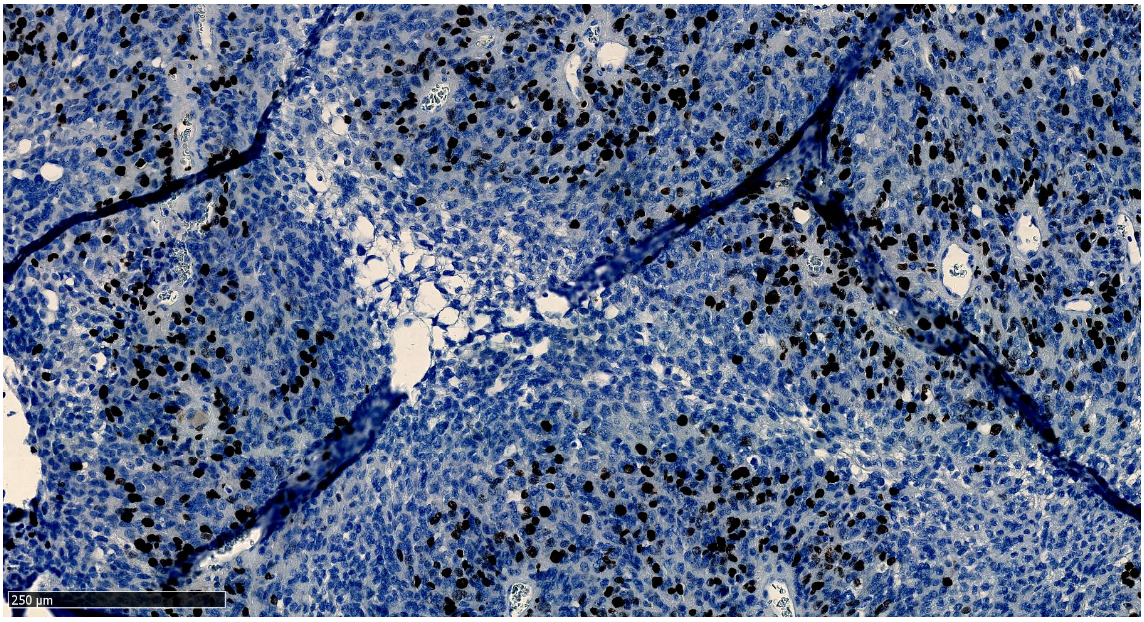

Figure S19. Case 19.

Case 19: Type 1 proliferative pattern, papillary meningioma, WHO grade III. “Clean” areas between hot spots (compared to case 18). Unfortunately, with artefactual folding and a rather thick slide. Total magnification ×100.

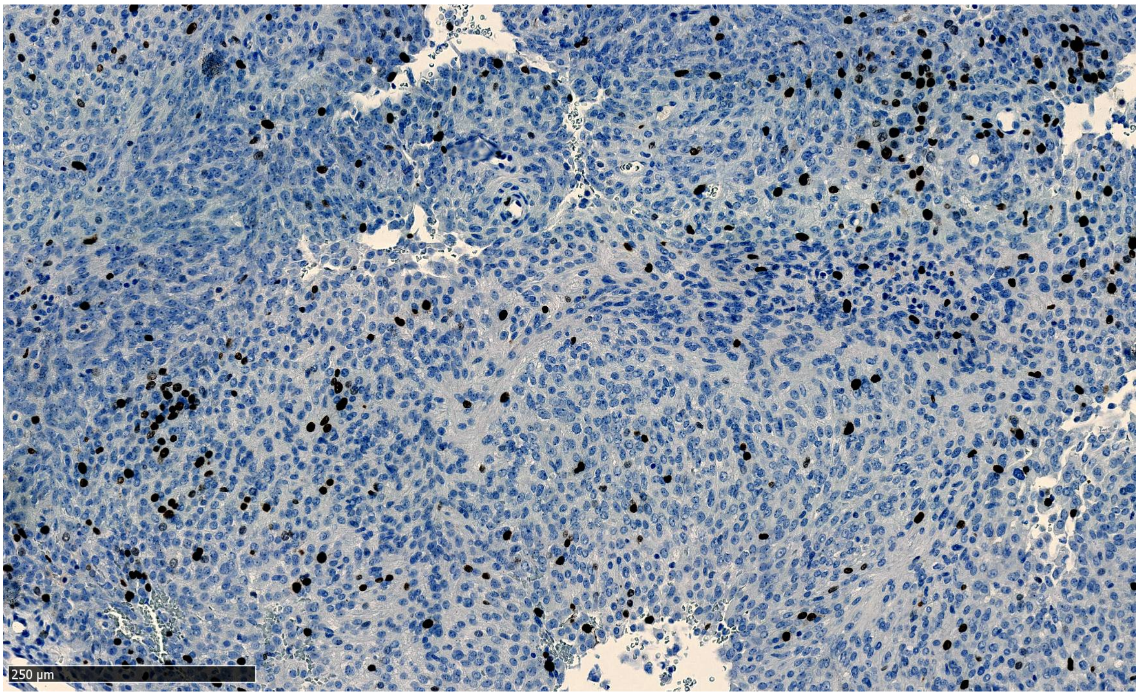

Figure S20. Case 20.

Case 20: Type 2 proliferative pattern, anaplastic meningioma, WHO grade III. Two hot spots in lower left and upper right corners on a relatively low proliferative background. Total magnification ×70.

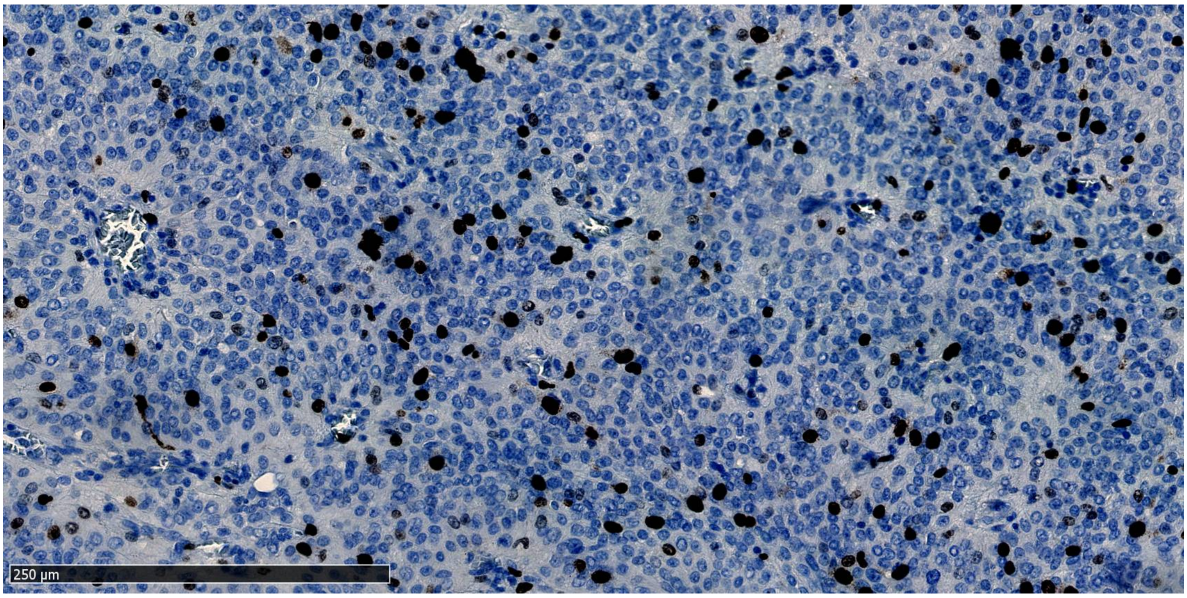

Figure S21. Case 21.

Case 21: Type 2 proliferative pattern, papillary meningioma, WHO grade III. Total magnification ×200.

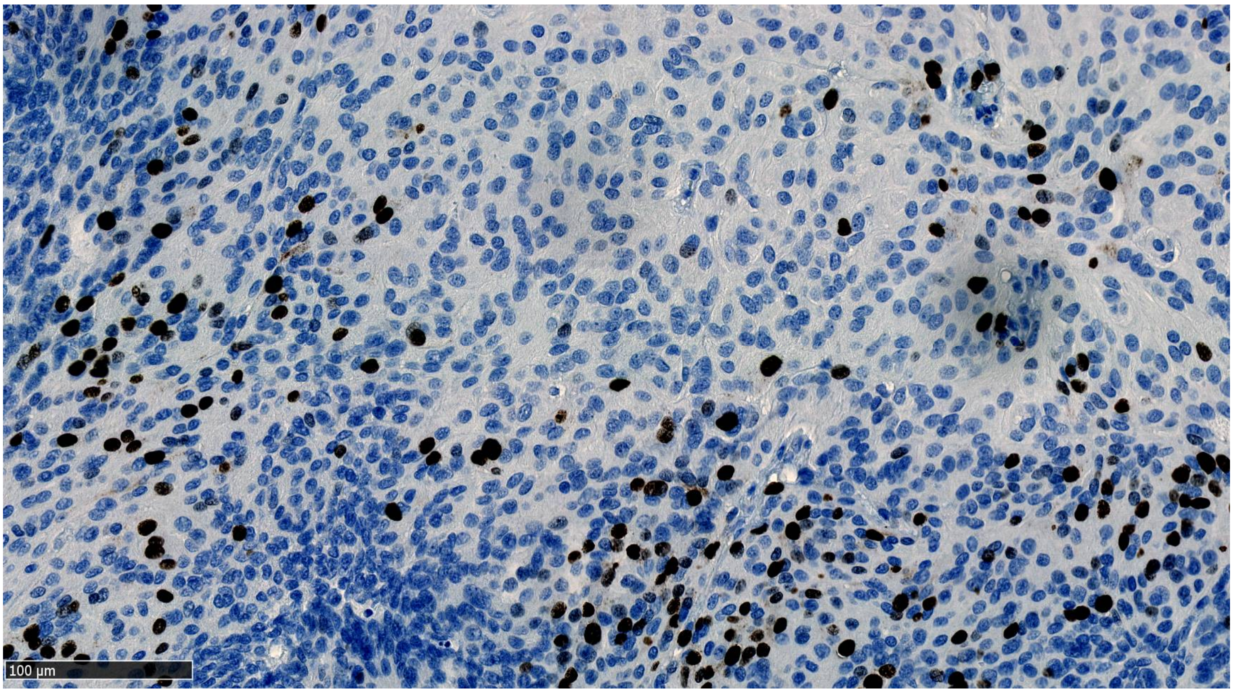

Figure S22. Case 22.

Case 22: Type 1 proliferative pattern, anaplastic meningioma, WHO grade III. Total magnification ×240.

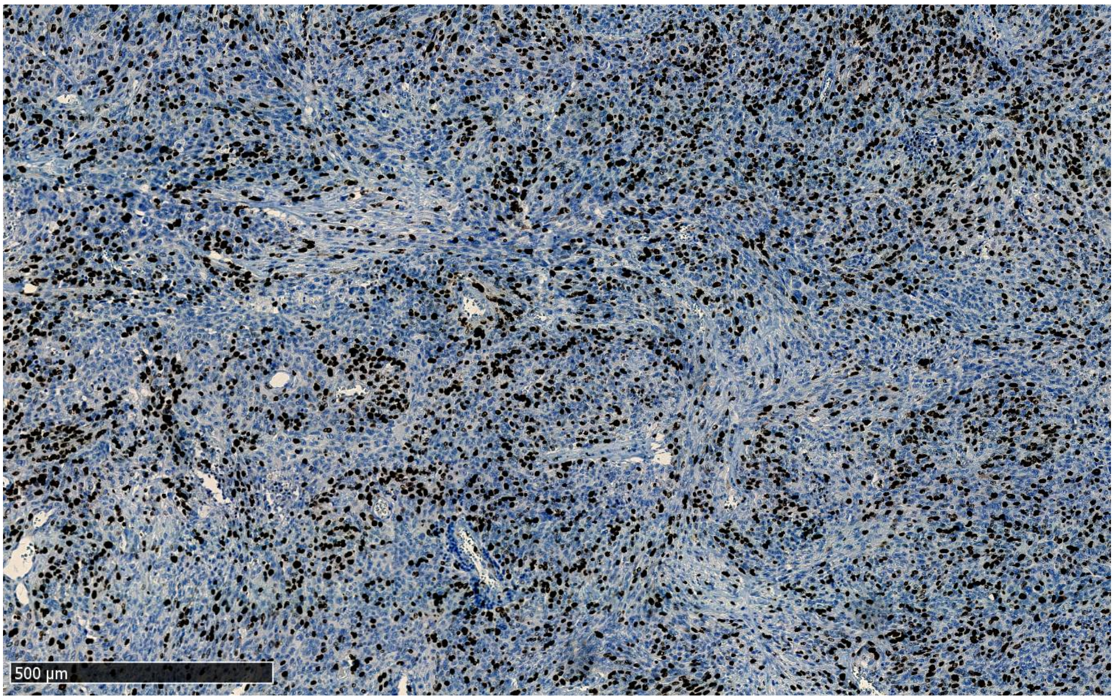

Figure S23. Case 23.

Case 23: Type 2 proliferative tumor, anaplastic meningioma, WHO grade III. Total magnification ×50.

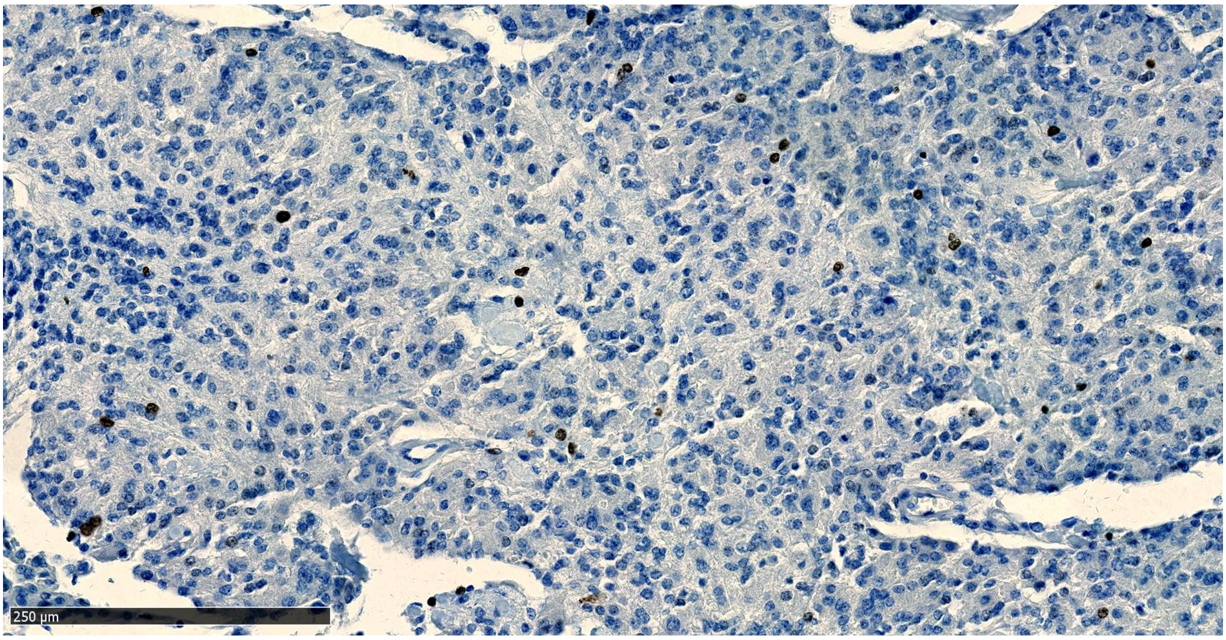

Figure S24. Case 24.

Case 24: Proliferative background level in type 2 proliferative pattern in a papillary meningioma, WHO grade III. Total magnification ×150.

© 2020 by the authors. Licensee MDPI, Basel, Switzerland. This article is an open access article distributed under the terms and conditions of the Creative Commons Attribution (CC BY) license (http://creativecommons.org/licenses/by/4.0/).
